# Systematic evaluation of Merkel cell carcinoma clinical practice guidelines using the AGREE II instrument

**DOI:** 10.1007/s00403-024-02853-0

**Published:** 2024-04-25

**Authors:** Deepak Lakshmipathy, Christian Fritz, Jacob Harris, Tejas Athni, Beatrice Go, Alvaro Moreira, Cerrene Giordano, Karthik Rajasekaran

**Affiliations:** 1 Department of Otorhinolaryngology–Head & Neck Surgery, University of Pennsylvania, 800 Walnut Street, 18th Floor, Philadelphia, PA 19107 USA; 2 Perelman School of Medicine, University of Pennsylvania, Philadelphia, PA USA; 3 Harvard Medical School, Harvard University, Boston, MA USA; 4 Department of Pediatrics, University of Texas Health Science Center at San Antonio, San Antonio, TX USA; 5 Department of Dermatology, University of Pennsylvania, Philadelphia, PA USA; 6 Leonard Davis Institute of Health Economics, University of Pennsylvania, Philadelphia, PA USA

**Keywords:** Merkel cell carcinoma, Clinical practice guidelines, Quality assurance, Statistics and research methods

## Abstract

Merkel cell carcinoma (MCC) is a rare type of skin cancer that requires a multidisciplinary approach with a variety of specialists for management and treatment. Clinical practice guidelines (CPGs) have recently been established to standardize management algorithms. The objective of this study was to appraise such CPGs via the Appraisal of Guidelines for Research and Evaluation (AGREE II) instrument. Eight CPGs were identified via systematic literature search following Preferred Reporting Items for Systematic Reviews and Meta-Analyses (PRISMA) criteria. Four appraisers trained in AGREE II protocols evaluated each CPG and deemed two CPGs as high quality, five as moderate quality, and one as low quality. Intraclass correlation coefficients (ICCs) were calculated to verify reviewer consistency as excellent, good, and moderate across four, one, and one domain, respectively. The majority of MCC CPGs are lacking in specifying stakeholder involvement, applicability, and rigor of development. The two high quality CPGs are from the Alberta Health Services (AHS) and the collaboration between the European Dermatology Forum, the European Association of Dermato-Oncology, and the European Organization of Research and Treatment of Cancer (EDF/EADO/EORTC). The EDF/EADO/EORTC CPG had the highest overall score with no significant deficiencies across any domain. An important limitation is that the AGREE II instrument is not designed to evaluate the validity of each CPG’s recommendations; conclusions therefore can only be drawn about each CPG’s developmental quality. Future MCC CPGs may benefit from garnering public perspectives, inviting external expert review, and considering available resources and implementation barriers during their developmental stages.

## Introduction

Merkel cell carcinoma (MCC) is an uncommon type of neuroendocrine tumor, accounting for a minority of skin cancers with approximately 0.5 cases per 100,000 persons [24]. Despite its rarity, it has a high rate of recurrence and can progress rapidly when left untreated. Often presenting as a painless nodule with varying color in the head and neck region, presentation can range in severity from localized, node negative disease to multiple metastatic lesions [33]. The pathophysiology of MCC is not completely understood but is thought to involve interplay between immunosuppression, Merkel cell polyomavirus, and ultraviolet radiation [8, 33]. The gold standard of diagnosis relies on a thorough history and physical followed by biopsy with dermatopathological evaluation. Further diagnostic testing with imaging is patient-specific and largely depends on their stage of disease [8]. Staging protocols are based on the eighth edition of the American Joint Committee on Cancer (AJCC) staging manual. Specifically, the primary tumor size (T), lymph node involvement (N), and existence of metastatic disease (M) are used in tandem to categorize MCC disease states between stages I through IV [1].

Management and outcomes are determined by staging. Generally, patients with lower stage disease receive more conservative treatment with more positive outcomes while patients with higher stage disease receive more aggressive treatment with less favorable outcomes [25]. Specific treatment also often varies between institutions and requires teamwork between multiple specialists. As a result, clinical practice guidelines (CPGs) have been developed to standardize management across different medical centers as well as systematically incorporate new treatment modalities into decision-making algorithms [31]. Rare pathologies like MCC have had a particularly high influx of new CPGs due to increased experience and patient volumes along with cumulative clinical trial data [3, 9, 12, 13, 22, 26–28]. Despite often being created by well-known experts in the field, the merit of CPGs is uncommonly appraised by objective criteria.

The Appraisal of Guidelines for Research and Evaluation (AGREE) II instrument is the second iteration of a tool developed more than two decades ago to specifically address this gap. It systematically evaluates CPGs over six quality domains with 23 specific key items to ensure that each CPG has internal and external validity [4, 7]. The tool was designed with multidisciplinary users in mind, allowing for facile implementation after a short training period. The strength of the instrument lies in its comparative power [4, 7]. Specifically, after multiple appraisers assign individual scores (based on a Likert scale) to each CPG, the strength of CPGs across specific domains can be compared [20]. Overall quality of each CPG can then be assessed and suggestions can be given regarding their referability or future development [4, 7].

With all this in mind, we generated the following research question: for patients with MCC, what CPGs on management exist and how can differentiating traits between higher and lower quality guidelines be used to raise the global standard of provided recommendations? We decided to answer this question using the AGREE II instrument given its success in evaluating CPGs for several other cutaneous pathologies across different patient populations [16, 21, 32]. Our aim is for guideline authors and methodologists to reference high quality CPGs when developing their own while simultaneously avoiding pitfalls of CPGs deemed as lower quality.

## Methods

### Systematic literature search

**Fig. 1:**
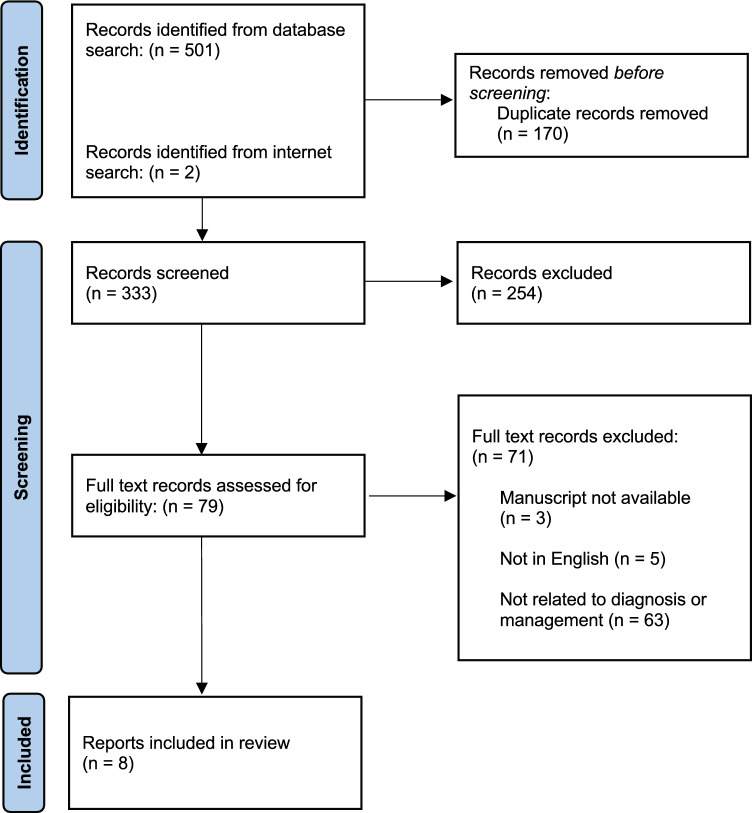
Flowchart showcasing systematic literature search and clinical practice guideline identification per Preferred Reporting Items for Systematic Reviews and Meta-Analyses (PRISMA) criteria

A systematic literature search was performed following the Preferred Reporting Items for Systematic Reviews and Meta-Analyses (PRISMA) outline in Fig. 1. MEDLINE via PubMed, Scopus, and Web of Science were used as source databases and were supplemented with internet searching. Search terms of “((merkel cell) AND (carcinoma* OR cancer*)) AND ((clinical* AND practice AND guideline*) OR guideline* OR consensus OR recommendation*)” were used alongside medical subject heading (MeSH) terms of “Carcinoma, Merkel Cell” and “Practice Guidelines as Topic.” Inclusion criteria consisted of original research articles with a primary focus on the management of MCC. Non-English literature without available full text copies and articles unrelated to MCC treatment were excluded. Eight relevant CPGs were identified after duplicate removal and title, abstract, and full-text screening.

### AGREE II evaluation

The eight selected CPGs were then evaluated by authors of this work (DL, CF, JH, TA) using the AGREE II instrument. Each author has previously completed formal appraiser training provided via the AGREE II manual and information listed on the website (<https://www.agreetrust.org/>). Evaluators assigned an individual score ranging from one (strongly disagree) to seven (strongly agree) for each CPG across the 23 key items and six overarching domains listed in Table 1.

**Table 1:** 23 Key items and six overarching domains comprising AGREE II instrument

Scope and purpose	
1	The overall objective(s) of the guideline is (are) specifically described
2	The health question(s) covered by the guideline is (are) specifically described
3	The population (e.g., patients, public) to whom the guideline is meant to apply is specifically described
Stakeholder involvement	
4	The guideline development group includes individuals from all relevant professional groups
5	The views and preferences of the target population (e.g., patients, public) have been sought
6	The target users of the guideline are clearly defined
Rigor of development	
7	Systematic methods were used to search for evidence
8	The criteria for selecting the evidence are clearly described
9	The strengths and limitations of the body of evidence are clearly described
10	The methods for formulating the recommendations are clearly described
11	The health benefits, side effects and risks have been considered in formulating the recommendations
12	There is an explicit link between the recommendations and the supporting evidence
13	The guideline has been externally reviewed by experts before its publication
14	A procedure for updating the guideline is provided
Clarity of presentation	
15	The recommendations are specific and unambiguous
16	The different options for management of the condition or health issue are clearly presented
17	Key recommendations are easily identifiable
Applicability	
18	The guideline describes facilitators and barriers to its application
19	The guideline provides advice and/or tools on how the recommendations can be put into practice
20	The potential resource implications of applying the recommendations have been considered
21	The guideline presents monitoring and/or auditing criteria
Editorial independence	
22	The views of the funding body have not influenced the content of the guideline
23	Competing interests of guideline development group members have been recorded and addressed

*AGREE II* Appraisal of Guidelines for Research and Evaluation

Once all of four appraisers’ ratings were collected and organized for each of the six domains, scaled domain scores were calculated via Microsoft Excel (Version 16.76; Microsoft Corporation) using the following formula:

$${\text{scaled}}\, {\text{domain}}\,{\text{score}}\,(\% ) = \frac{{{\text{obtained}}\,{\text{score}} - {\text{minimum}}\,{\text{ possible}}\,{\text{ score}}}}{{{\text{maximum }}\,{\text{possible}}\,{\text{ score}} - {\text{minimum }}\,{\text{possible}}\,{\text{score}}}} \times 100$$

Obtained score, minimum possible score, and maximum possible score represent the sum total of all actual appraiser scores, minimum possible appraiser scores (i.e. one point per item per appraiser), and maximum possible appraiser scores (i.e. seven points per items per appraiser) across each item of each domain, respectively. Associated means and standard deviations (SD) were also calculated across each CPG and domain using the same software. Overall CPG quality appraisals of high, moderate, or low were then assigned if ≥5, 3–4, or ≤2 domains had scaled domain scores of ≥ 60%, respectively.

### Interrater reliability assessment

Intraclass correlation coefficients (ICC) and associated 95% confidence intervals (CI) were also calculated via RStudio (Version 2023.06.1+524; RStudio Team) to assess interrater reliability. Using an established consensus as reference, excellent, good, moderate, and poor interrater reliability were defined by ICC thresholds of >0.90, 0.75–0.90, 0.50–0.75, <0.50, respectively [19].

## Results

### Selected CPGs

The eight MCC CPGs selected from the systematic literature search are characterized in Table 2 and arise from the following organizations: German Society of Dermatology (DDG), Alberta Health Services (AHS), Spanish Academy of Dermatology and Venereology (AEDV), European Dermatology Forum, European Association of Dermato-Oncology, European Organization of Research and Treatment of Cancer (EDF/EADO/EORTC), Danish MCC Expert Group (DEG), National Comprehensive Cancer Network (NCCN), Society for Immunotherapy of Cancer (SITC), and Italian Association of Medical Oncology (AIOM). All were published or updated within the past 5 years, are intended for a multidisciplinary user base, utilize expert consensus and literature review as sources of information, and provide suggestions for the management of MCC. All CPGs were from either North America or Europe.

**Table 2:** Selected clinical practice guidelines on Merkel cell carcinoma and their associated general characteristics

First author	Development group	Abbreviation	Region of origin/focus	Funding	Intended users	Evidence base	Guideline content
Becker [3]	German Society of Dermatology	DDG	Germany	Project Deal	Multidisciplinary	Expert consensus, literature review	Diagnosis and treatment of MCC
Craighead [9]	Alberta Health Services	AHS	Canada	AHS	Multidisciplinary	Expert consensus, literature review	Diagnosis and treatment of MCC
Doval [12]	Spanish Academy of Dermatology and Venereology	AEDV	Spain	AEDV	Multidisciplinary	Expert consensus, literature review	Diagnosis and treatment of MCC
Gauci [13]	European Dermatology Forum, the European Association of Dermato-Oncology, and the European Organization of Research and Treatment of Cancer	EDF/EADO/EORTC	Europe	–	Multidisciplinary	Expert consensus, literature review	Diagnosis and treatment of MCC
Naseri [22]	Danish MCC Expert Group	DEG	Denmark	–	Multidisciplinary	Expert consensus, systematic literature review	Diagnosis and treatment of MCC
Schmults [26]	National Comprehensive Cancer Network	NCCN	United States	NCCN Foundation	Multidisciplinary	Expert consensus, systematic literature review	Diagnosis and treatment of MCC
Silk [27]	Society for Immunotherapy of Cancer	SITC	United States	SITC	Multidisciplinary	Expert consensus, literature review	Immunotherapy for treatment of nonmelanoma skin cancer
Spada [28]	Italian Association of Medical Oncology	AIOM	Italy	Merck, Pfizer	Multidisciplinary	Expert consensus, literature review	Diagnosis and treatment of MCC

*MCC* Merkel cell carcinoma

### Quality designations

The scaled domain scores stratified by each CPG and AGREE II domain are shown in Table 3 alongside their overall quality appraisal. Domains 4 (clarity of presentation) and 6 (editorial independence) had the highest overall scores across the eight CPGs with 90.10 ± 8.98% and 88.80 ± 5.33%, respectively. All CPGs appeared to be lacking in domains 2 (stakeholder involvement), 3 (rigor of development), and 5 (applicability) with mean overall scores of 49.65 ± 10.12%, 58.98 ± 18.47%, and 56.64 ± 17.55%, respectively. The EDF/EADO/EORTC guideline had the highest mean overall score (79.80%) across the six domains while the DDG guideline had the lowest (58.62%). Based on aforementioned criteria, the AHS and EDF/EADO/EORTC guidelines were deemed as high quality; DDG, AEDV, NCCN, SITC, and AIOM guidelines were categorized as moderate quality. The DEG guideline was the only guideline appraised to be low quality.

**Table 3:** AGREE II instrument scaled domain scores, mean overall scores, and quality appraisals for each MCC CPG

Guideline	Domain 1: Scope and purpose (%)	Domain 2: Stakeholder involvement (%)	Domain 3: Rigor of development (%)	Domain 4: Clarity of presentation (%)	Domain 5: Applicability (%)	Domain 6: Editorial independence (%)	Mean overall score	Quality appraisal
DDG	27.78	31.94	22.92	93.06	78.13	97.92	58.62	Moderate
AHS	97.22	61.11	64.06	97.22	36.46	93.75	74.97	High
AEDV	88.89	50.00	84.90	95.83	38.54	87.50	74.28	Moderate
EDF/EADO/EORTC	81.94	63.89	75.00	86.11	82.29	89.58	79.80	High
DEG	45.83	48.61	48.96	86.11	45.83	89.58	60.82	Low
NCCN	48.61	43.06	63.02	97.22	59.38	83.33	65.77	Moderate
SITC	66.67	45.83	55.21	94.44	65.63	87.50	69.21	Moderate
AIOM	69.44	52.78	57.81	70.83	46.88	81.25	63.17	Moderate
Mean ± SD	65.80 ± 23.71	49.65 ± 10.12	58.98 ± 18.47	90.10 ± 8.98	56.64 ± 17.55	88.80 ± 5.33	65.80 ± 23.71	

*AGREE II* Appraisal of Guidelines for Research and Evaluation, *MCC* Merkel cell carcinoma, *CPG* clinical practice guideline, *DDG* German Society of Dermatology, *AHS* Alberta Health Services, *AEDV* Spanish Academy of Dermatology and Venereology, *EDF/EADO/EORTC* European Dermatology Forum, the European Association of Dermato-Oncology, and the European Organization of Research and Treatment of Cancer, *DEG* Danish MCC Expert Group, *NCCN* National Comprehensive Cancer Network, *SITC* Society for Immunotherapy of Cancer, *AIOM* Italian Association of Medical Oncology

### Interrater reliability

ICCs and associated 95% CIs for each of the six domains are shown in Table 4. Using the thresholds described earlier, domains 1 (scope and purpose), 2 (stakeholder involvement), 3 (rigor of development), and 5 (applicability) all demonstrated excellent interrater reliability. Domain 6 (editorial independence) had good interrater reliability and domain 4 (clarity of presentation) had moderate reliability. No domains had poor interrater reliability. ICCs across all domains were statistically significant as no CIs included the null hypothesis ICC value of 0.

**Table 4:** Intraclass correlation coefficients for each of the six AGREE II domains

AGREE II domain	ICC	95% CI
Domain 1: Scope and purpose	0.96	[0.77, 1.00]
Domain 2: Stakeholder involvement	0.99	[0.94, 1.00]
Domain 3: Rigor of development	0.98	[0.94, 1.00]
Domain 4: Clarity of presentation	0.74	[0.53, 0.99]
Domain 5: Applicability	0.94	[0.70, 1.00]
Domain 6: Editorial independence	0.83	[0.11, 1.00]

*AGREE II* Appraisal of Guidelines for Research and Evaluation, *ICC* Intraclass correlation coefficient, *CI* confidence interval

## Discussion

### Representativeness of CPGs

The systematic search strategy described above (Fig. 1) alongside the CPGs’ varied authors, collaborative organizations, and countries of origin (Table 2) highlight that the included studies represent most, if not all, MCC CPGs [3, 9, 12, 13, 22, 26–28]. It should also be noted multiple weeks went into refining final search terms and how each guideline went through two rounds of screening (first by title and abstract and then by full text) with voting at the end of each stage from multiple authors (DL, CF, KR) to ensure database searches were comprehensive and selected CPGs were relevant. Assessment of CPGs were similarly rigorous with reviewers undergoing weeks of AGREE II training prior to guideline appraisal, all reviewer ratings undergoing quality control against objective criteria outlined in the AGREE II manual, and mathematical calculations being independently verified by two authors (DL and CF). Although no financial resources were required, the time invested by specialized reviewers was pivotal in establishing presented findings as representative and trustworthy. Moreover, the CPGs likely incorporate the most modern clinical evidence regarding MCC. All CPGs utilize the most recent (eighth) edition of the AJCC staging manual [1, 3, 9, 12, 13, 22, 26–28]. Some CPGs explicitly list themselves as updates of prior versions, highlighting which evidence-based changes were made [3, 9, 13, 26]. Furthermore, the SITC guideline exclusively deals with one of the newest treatment modalities of MCC–immunotherapy [27]. The role of immunotherapy in MCC began receiving serious attention over the past decade when clinical trials began reporting preliminary but promising data [10, 17, 23, 30]. Altogether, the collection of CPGs included in this article are up-to-date representatives of the body of literature and will likely serve as frameworks for future CPGs.

### Domain strengths

Table 3 demonstrates that domains 4 (clarity of presentation) and 6 (editorial independence) are strong across almost every CPG. High clarity of presentation implies that main recommendations from each guideline are well demarcated and that multiple treatment options are explicitly presented. This is an important strength in the context of MCC, as dermatologists, otolaryngologists, plastic surgeons, and medical and radiation oncologists often work alongside each other when caring for these patients. For instance, during tumor boards, multiple specialists can easily reference these CPGs and quickly understand specialty-specific decision-making when deciding on the next step of MCC treatment. High editorial independence implies that neither funding nor conflicts of interest significantly altered each CPG’s recommendations. This ensures that management algorithms are dictated by evidence-based outcomes rather than financial incentives. Focus on best clinical practice is critical in MCC given its aforementioned aggressiveness and high rate of recurrence; accurate diagnosis with targeted treatment can also help limit the high healthcare costs imposed on this patient population [5, 6, 18, 29, 33, 34].

### Domain weaknesses

The selected CPGs are noticeably weaker across domains 2 (stakeholder involvement), 3 (rigor of development), and 5 (applicability). Upon closer inspection of individual appraiser values, the low scaled domain score for stakeholder involvement mainly arose from low item 5 scores (Table 1). This suggests that most CPG authors struggled to include perspectives from the target population. The exact reasons behind this are unclear but likely stem from the small patient population and costliness of gathering public opinion [2, 24]. Regardless, patient views and preferences should be sought to help guide CPG authors on what topics should be addressed and bolster recommendations based on limited supporting evidence. Guideline authors should consider involving other skin cancer patients if recruiting MCC patients proves difficult, as this alternative is better than omitting patient perspectives altogether. However, despite domain 2’s low overall scaled score, most CPGs did well in including relevant professionals and defining target users during guideline creation.

The low rigor of development (domain 3) value followed a similar trend in that very low item 13 and low 14 scores (Table 1) brought down the overall scaled domain score. Regarding item 13, most, if not at all, CPGs had a distinct absence of any external expert review prior to publication. This is an important flaw in validity, as lack of outside opinion could cause groupthink amongst CPG authors and subsequently limit generalizability [11]. More variability was present among item 14 as half of the CPGs provided a clear protocol for incorporating new updates into their guidelines while the remaining half failed to mention it altogether. Authors who are interested in contributing to new CPGs would benefit from including detailed update instructions. The CPGs did well in other aspects of domain 3 such as: efforts to include systematic searches, inclusion/exclusion criteria and critical evaluation for evidence, and methods and expert appraisal behind recommendation formulation. This suggests that those domain features are ubiquitous among higher quality CPGs.

In contrast to the two previously discussed domains, the low applicability (domain 5) value was due to uniformly low scores across all domain items (items 18–21, Table 3). These items are all closely linked to the topic of implementing each CPG’s recommendations (i.e. facilitators, barriers, resource limitations, and periodic auditing). Issues in this domain may stem from the physician authors’ limited exposure to the medical supply chain, non-patient-related clinical tasks, and quality assurance metrics. Therefore, future CPG development boards may benefit by including a health economist who can give input about those realms [15].

### Global quality of guidelines for MCC

In terms of overall appraisals, most MCC CPGs were determined to be of moderate quality or higher (Table 3). The EDF/EADO/EORTC guideline had the highest overall score with no significant deficiencies across any domain. Although the AHS guideline was also high quality, it was relatively lacking in domain 5 (applicability). The authors of this study therefore recommend updaters of current CPGs and authors of future ones reference the EDF/EADO/EORTC as a framework. It should be noted that the high quality guidelines (EDF/EADO/EORTC and AHS) are less widely known than some of their moderate quality counterparts (i.e. NCCN), showcasing how a guideline’s popularity does not necessarily dictate its developmental excellence [14]. The DEG guideline was the only low quality CPG, lacking in most of the six overarching domains. Interestingly, it did perform relatively well in domains 4 (clarity of presentation) and 6 (editorial independence). This illuminates how low quality guidelines may not only serve as a weaker example to learn from but also provide positive value in certain aspects.

### Validity of findings

The ICC values shown in Table 4 validate the reliability of this systematic evaluation’s findings. Having excellent interrater reliability across four of the six domains as well as good and moderate values for the remaining two, showcase that each appraiser (DL, CF, JH, TA) evaluated the CPGs with a near identical interpretation of the AGREE II instrument. This implies both that the training provided online by the AGREE Next Steps Consortium is comprehensive and that having the recommended number of appraisers is effective in eliminating biases between raters [4, 7]. The excellent ICCs present among domains 1 (scope and purpose), 2 (stakeholder involvement), 3 (rigor of development), and 5 (applicability) suggest that these topics require no additional discussion between appraisers. The good ICC value for domain 6 (editorial independence) may have stemmed from some mild ambiguity in how appraisers define funding or competing interests as influential. Thus, future appraisers may marginally benefit from additional discussion on this topic before grading CPGs. The moderate ICC for domain 4 (clarity of presentation) might have arisen from minor subjective bias in how each evaluator prefers data presentation within CPGs. Evaluators should consequently consider establishing objective checklists between themselves on what is considered “clear” beforehand.

### Study limitations

As with any study, it is important to note the associated limitations. Beginning with the literature search, there is possible publication bias from non-published negative data and lack of public access to institution-specific MCC treatment protocols. Excluding five non-English articles also may have introduced minor amounts of selection bias. Another important limitation lies with inherent subjectiveness of the AGREE II instrument. Despite the appraisers being specifically trained to use the tool and having the electronic manual on-hand while evaluating CPGs, the use of a Likert scale relies on subjective judgment to differentiate between point values [20]. The impact of this subjectivity was likely mitigated by the strong interrater reliabilities described above but cannot be eliminated altogether. The scaled domain scores referenced when creating overall quality impressions are also unevenly subject to interrater fluctuations. For example, domain 6 (editorial independence) contains only two items while domain 3 (rigor of development) has eight. So, despite having four reviewer scores for each item, the scaled domain score for domain 6 has six fewer data points in its calculation than that for domain 3. Finally, it should be noted that the AGREE II instrument is not designed to evaluate the validity of each CPG’s recommendations. The overall quality assessments are consequently limited to the developmental methodology, presentation, and clarity of each CPG. Even low quality CPGs that lack well-defined research questions, systematic literature searches, and/or critical appraisal of evidence should therefore not be completely discounted. Especially if their recommendations touch upon topics with limited available data (i.e. new types of immunotherapy), expert consensus can still serve as evidence and provide valuable clinical pearls for management.

In conclusion, the majority of CPGs for MCC are of acceptable quality with the potential to standardize management of the disease. We recommend use of the EDF/EADO/EORTC guideline as a developmental framework with the AHS guideline as a valid alternative. These suggestions are based on an objective, validated measurement tool with a high degree of interrater consistency. Future updates or new CPGs may benefit from garnering patient and public perspectives, inviting external expert review, and considering available resources and implementation barriers during their creation.

## Data Availability

All data supporting the findings of this study are available within the paper.
